# A Review of Characteristics of Bio-Oils and Their Utilization as Additives of Asphalts

**DOI:** 10.3390/molecules26165049

**Published:** 2021-08-20

**Authors:** Ran Zhang, Zhanping You, Jie Ji, Qingwen Shi, Zhi Suo

**Affiliations:** 1 School of Civil and Transportation Engineering, Beijing University of Civil Engineering and Architecture, Beijing 100044, China; zhangran@bucea.edu.cn (R.Z.); jijie@bucea.edu.cn (J.J.); shiqingwen1994@163.com (Q.S.); 2 Beijing Advanced Innovation Center for Future Urban Design, Beijing 100044, China; 3 Beijing Collaborative Innovation Center for Energy Conservation & Emission Reduction and Sustainable Urban-Rural Development, Beijing 100044, China; 4 Department of Civil and Environmental Engineering, Michigan Technological University, 1400 Townsend Drive, Houghton, MI 49931, USA; zyou@mtu.edu

**Keywords:** biomass, bio-oil, bio-oil modified asphalt, chemistry, molecules, properties

## Abstract

Transforming waste biomass materials into bio-oils in order to partially substitute petroleum asphalt can reduce environmental pollution and fossil energy consumption and has economic benefits. The characteristics of bio-oils and their utilization as additives of asphalts are the focus of this review. First, physicochemical properties of various bio-oils are characterized. Then, conventional, rheological, and chemical properties of bio-oil modified asphalt binders are synthetically reviewed, as well as road performance of bio-oil modified asphalt mixtures. Finally, performance optimization is discussed for bio-asphalt binders and mixtures. This review indicates that bio-oils are highly complex materials that contain various compounds. Moreover, bio-oils are source-depending materials for which its properties vary with different sources. Most bio-oils have a favorable stimulus upon the low temperature performance of asphalt binders and mixtures but exhibit a negative impact on their high-temperature performance. Moreover, a large amount of oxygen element, oxygen-comprising functional groups, and light components in plant-based bio-oils result in higher sensitivity to ageing of bio-oil modified asphalts. In order to increase the performance of bio-asphalts, most research has been limited to adding additive agents to bio-asphalts; therefore, more reasonable optimization methods need to be proposed. Furthermore, upcoming exploration is also needed to identify reasonable evaluation indicators of bio-oils, modification mechanisms of bio-asphalts, and long-term performance tracking in field applications of bio-asphalts during pavement service life.

## 1. Introduction

Since petroleum is a finite resource that cannot be replenished, more researchers are exploring novel methods to minimize the utilization of virgin petroleum asphalt binder in road construction [1,2]. Likewise, the search for ecologically acceptable materials is gaining traction among scholars [3,4,5]. Currently, various biomass products, such as waste cooking oil (WCO) [6,7], animal droppings [8], wood waste [3], corn stover [9], and others, are thrown out or cannot be used efficiently, resulting in environmental degradation. Sixty percent of all biomass resources are waste agricultural commodities. In China, a tremendous amount of agricultural straw is produced every year; however, the majority of it exists in waste streams to be burnt, showing little inherent value, which adds significantly to air pollution and, as a result, can compromise traffic safety. Alternatively, biomass sources, which are abundant and cheap, can be recycled to produce bio-oils [10,11].

Employing bio-oils derived from various biomass sources to effectively substitute or amend petroleum asphalt is an innovative method to cut down the use of new petroleum asphalt while also producing ecologically beneficial products. Bio-oil has already gained popularity amongst renewable energy sources due to its benefits, including the existence of a wide variety of biomass sources, high yield, and cheap cost [12,13,14].

Bio-oils are a type of renewable substance made from biomass sources [15]. Various processes can be used to make bio-oils. Pyrolysis (PRL) and hydrothermal liquefaction (HTL) have become the two most common methods for producing plant-based bio-oils and animal waste-based bio-oils, respectively [16]. The pyrolysis processes are classified as fast PRL, slow PRL, flash PRL, and traditional PRL based on temperature and residence time [17]. Among these, fast pyrolysis has become the most common method applied in a large number of studies because of its favorable aspects, such as high yield, simplicity, and cheap cost. The biomass sources are degraded into vapors and chars throughout the manufacturing process, and the vapors are subsequently concentrated to produce gas and liquid. Following this procedure, three primary composites are produced: bio-chars, bio-gases, and bio-liquids also known as bio-oils [18,19]. Due to the difference in processing conditions, the properties of bio-oils may be affected by preparation technologies. However, the production of bio-oils is not the focus of this review paper, so it will not be described in detail in the text.

Numerous recent studies have reported basic characteristics of bio-oils and fundamental features of asphalt binders modified with bio-oils [7,12,14,20,21]. Many bio-oils have high asphalt modifying capability, and the effectiveness of bio-asphalt is mostly dependent on the biomass source. Bio-oils, such as those derived from waste wood and swine manure, can increase the low-temperature fracture resistance and workability of asphalt, but they can also reduce its high-temperature qualities [22,23]. Swine manure-based bio-asphalts are more resistant to ageing than plant-based bio-asphalts [24]. The great variabilities in the characteristics of bio-oils as well as some unsatisfactory properties of bio-asphalt binders and mixtures have become the important influencing factors in restricting the application of bio-oils in asphalts. Therefore, many scholars have also focused on improving the performance of bio-asphalts by adding other additives, such as SBS, nanomaterials, and others [2,12,25].

The purpose of this research is to deliver a review of the characteristics of bio-oils and their utilization as additives of asphalts. Specifically, several summaries are presented in this paper: (1) characteristics of bio-oils; (2) properties estimation of bio-oil modified asphalt binders; (3) performance assessment of bio-oil modified asphalt mixture; and (4) performance optimization of bio-oil modified asphalt binders/mixtures.

## 2. Review Methodology

**Figure 1:**
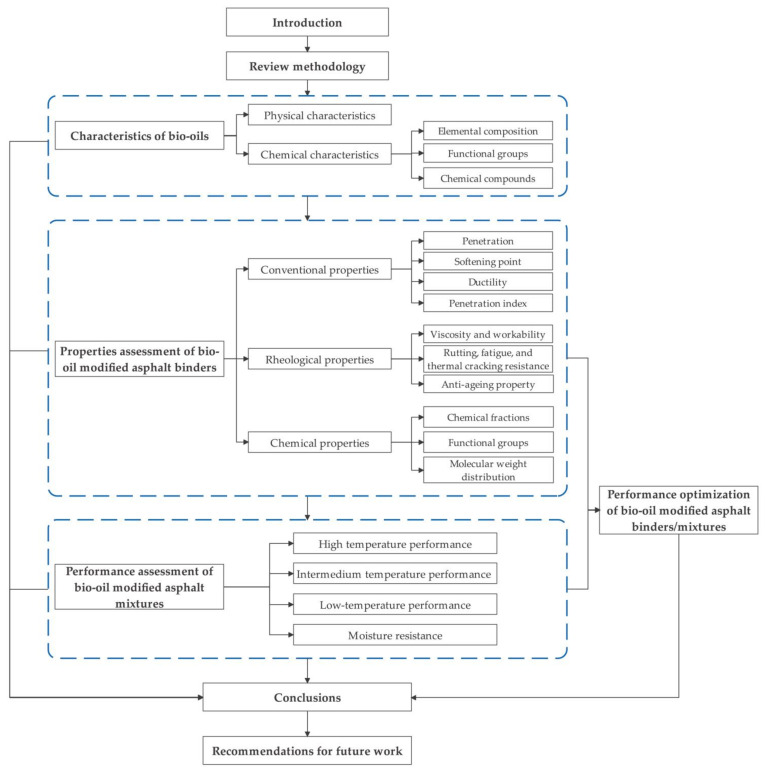
The flowchart of this review.

Currently, bio-oil modified asphalts are not being widely applied in pavement engineering. Unsatisfactory performance is the critical factor restricting its application, and most of the current research focuses on the performance of various bio-oils and bio-based asphalts. Motivated by this, the objective of this paper is to provide a review of the characteristics of various bio-oils, performance assessment, and performance optimization of bio-oil modified asphalt binders/mixtures. First, the physical and chemical properties of bio-oils generated from various biomass materials are characterized. Next, the utilization of bio-oils as additives of asphalts are reviewed. The performance of bio-oil modified asphalt binders and mixtures are evaluated, and then their performance optimization is reviewed. Finally, the conclusions are presented and future work is recommended. This paper provides direction for future research and has important theoretical guiding significance for promoting the practical application of bio-oils in pavement engineering. A flowchart of this review is shown in Figure 1.

**Figure 2:**
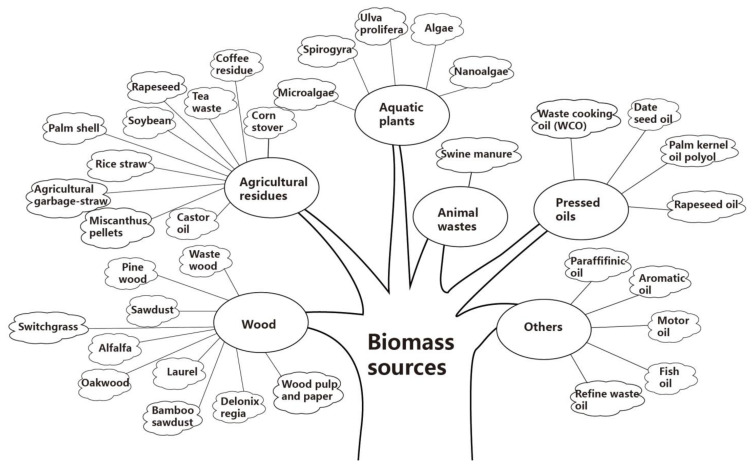
Various biomass sources involved in this review.

Bio-oils are derived from various biomass materials, including wood, agricultural residues, aquatic plants, animal wastes, pressed oils, and others [16]. The bio-oils involved in this review cover as many types as possible based on a large body of research. The specific biomass sources are shown in Figure 2.

## 3. Characteristics of Bio-Oils

### 3.1. Physical Characteristics

**Figure 3:**
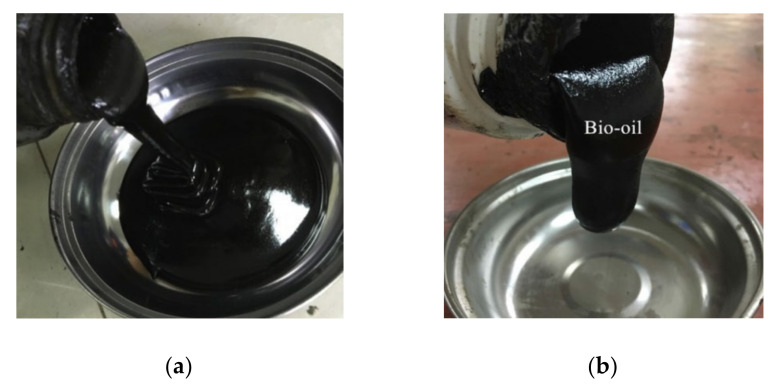

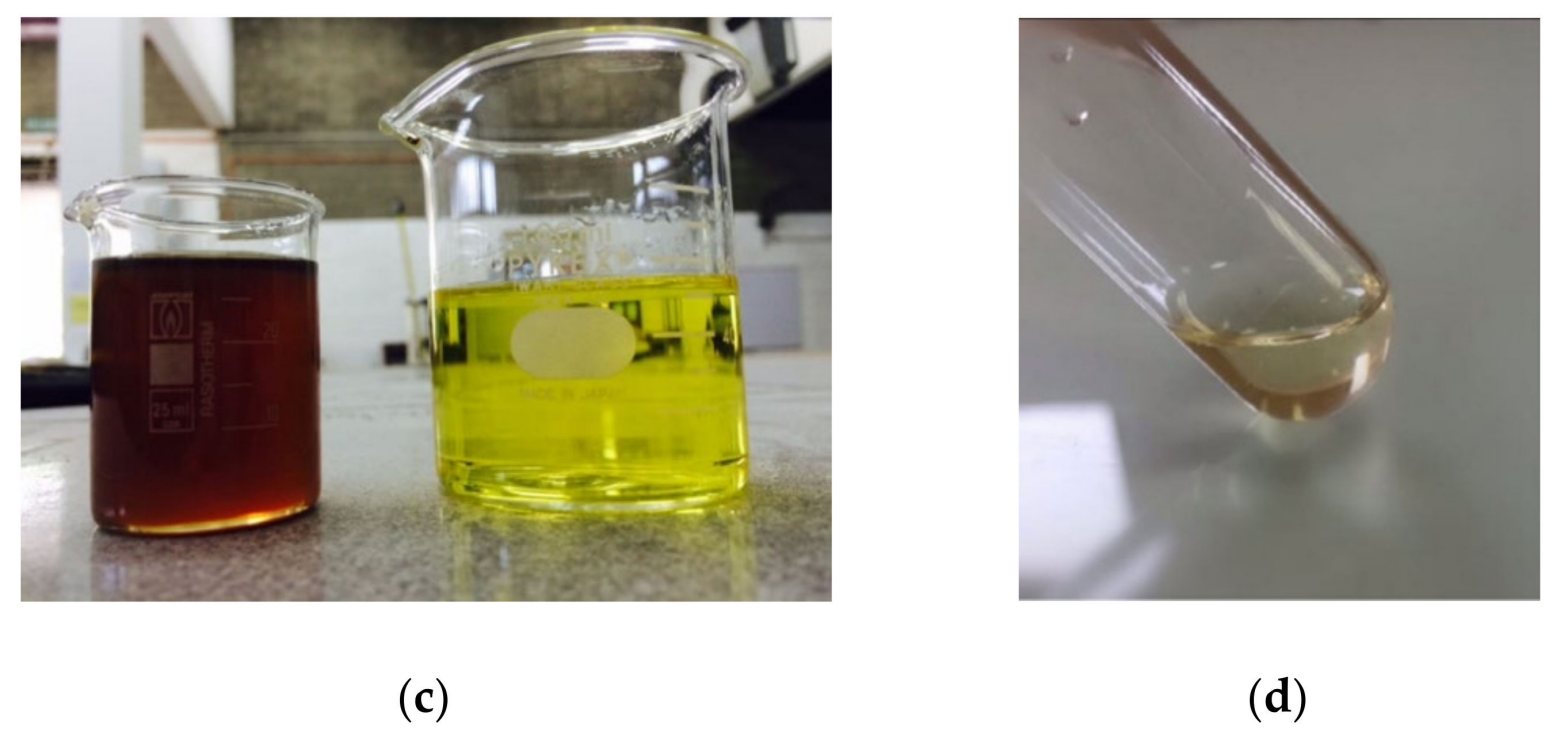
Bio-oils derived from various sources: (**a**) Waste wood [15]; (**b**) Corn stover [41]; (**c**) Waste cooking oil [42]; (**d**) Date seed oil [43].

At room temperature, bio-oils are free-flowing organic liquids [26,27,28]. Many biomass sources, such as waste wood [25,29], corn stover [30], animal wastes [31,32], rapeseed and soybeans [33,34], tea and coffee residue [35,36], microalgae [14], urban yard waste [37], and other biomass sources are used to make bio-oils. Various bio-oils are shown in Figure 3. Bio-oils are substances for which its characteristics differ based on the biosource. Since bio-oils include many organic acids, the pH of bio-oils has often been found to be between 2.5 and 3.5 [18,19]. Bio-oils have a moisture content ranging from 15 to 30%, and their viscosity values vary widely depending on the raw material and pyrolysis (PRL) conditions, ranging from 35 to 1000 cP at 40 °C [38]. Based on previous studies, the basic physical characteristics of bio-oils derived from softwood, waste wood, and other biomass sources are summarized and presented in Table 1 [18,19,20, 38,39,40].

### 3.2. Chemical Characteristics

#### 3.2.1. Elemental Composition

The elemental composition of bio-oils from various sources is summarized and presented in Table 2. It is evident that the four-element amounts within petroleum asphalt and bio-oils are different. In comparison to petroleum asphalt binders, the bio-oils have significantly higher oxygen (O) element and less carbon (C) element. For example, the O element amount in petroleum asphalt binder 1 is 1.33%, while it is as high as 45.19% in switchgrass bio-oil. That is an important reason for why bio-oils are prone to ageing. Moreover, the four-element amounts among bio-oils vary with biomass sources, a fact which is clearly reflected in the difference between the swine waste-based bio-oil and plant-based bio-oils. The element amount affects the functional groups and polar groups in the binder, which results in the difference in physical and chemical characteristics among bio-oils.

#### 3.2.2. Functional Groups

The functional groups of different bio-oils have been studied by Fourier transform infrared spectroscopy (FTIR), as summarized in Table 3. The bio-oils exhibit different functional groups from petroleum asphalt. Petroleum asphalt exhibits a large amount of C-H bending. However, there is a lot of S=O and C=O stretching and O-H stretching in waste wood bio-oil [20] and C-O stretching, C-H stretching, and O-H stretching in spirogyra bio-oil [54]. Oxygen-containing functional groups may contribute to the ageing of bio-oils. Similarly, plenty of aromatic compounds, ethers, and alkanes are contained in bio-oils. Moreover, it was found that the relative yield of phenols, ethers, alcoholic, and carboxylic in pine waste bio-oil was affected by the PRL temperature varying from 350–600 °C [55]. Cao et al., also noted that the composition of petroleum asphalt was dominated by hydrocarbons and had a higher saturation, whereas the composition of bio-oil was complex, mainly including hydrocarbons, esters, and aromatic hydrocarbons. This contributes to the difference in properties between petroleum asphalt and bio-oil modified asphalts. However, some functional groups in sawdust bio-oil were similar to petroleum asphalt, which may explain the better compatibility between bio-oil and petroleum asphalt [53].

#### 3.2.3. Chemical Compounds

In order to obtain the compounds of bio-oils, Gas Chromatography-Mass Spectrometry (GC-MS) equipment was utilized by researchers to analyze bio-oils generated from different sources. The test results of GC-MS for bio-oils are displayed in Table 4. It is understandable that bio-oils are highly complex materials that contain various compounds. The main compounds in bio-oils include acids, esters, phenolic compounds, and derivatives. Waste wood bio-oil mainly contains 2-Methoxy-4-methylphenol, naphthalene, and 2-methoxyphenol [21]. There is mainly l-polyglucose, caproic acid, and *o*-methoxyphenol in pine sawdust bio-oil [56] and 3,4,5-Tri-methyl pyrazole, 2,3,4-Trimethyl-d-xylose, and 1-*H* imidazole1,2,4,5-tetramethyl in spirogyra bio-oil [54]. In addition to the biomass source, the processing technology of bio-oil also has a great influence on its chemical compounds. For example, there is a difference in chemical compounds in Ulva prolifera bio-oil produced by liquefaction over the parent HY catalyst and over 8% Fe/HY catalyst [57].

Furthermore, fractional distillation has been frequently applied to separate the components of bio-oil. Moisture remains in the lighter fraction, oxygenated components and aromatic compounds occur in the light to intermediate fractions, and phenols exist in the heavy fraction [58]. Heavy bio-oils are commonly utilized for boiler burning and paving materials, whereas light bio-oils are often used for refining bio-gasoline, biodiesel, and other fuels. Many resin and polar constituents are included in bio-oils, which may be the main reason that they exhibit a stronger adherence to aggregates of bio-asphalt [21].

## 4. Properties Assessment of Bio-Oil Modified Asphalt Binders

An increasing number of researchers have looked into bio-oils as an approach to modify asphalt. Bio-oils have excellent compatibility with petroleum asphalt according to prior investigations. Therefore, the preparation of bio-oil modified asphalts is simple. Generally, bio-oil modified asphalts, referred to as bio-asphalts, are obtained by blending bio-oils and asphalt binders with different mixing temperature, mixing time, shear rate, and so on [20,42,59]. Many scholars have looked at the qualities of bio-asphalts in earlier studies.

### 4.1. Conventional Properties

#### 4.1.1. Penetration

The conventional qualities of bio-asphalts are generally described in terms of penetration, softening point, and ductility. When the binder is softer, the lesser the consistency, and the higher the penetration. As shown in Table 5, test results by He et al. [60], Li et al. [61] and Rasman et al. [62] showed the difference in penetration between base binders and bio-asphalts. As the bio-oil content increased, the penetration of asphalt binders enhanced gradually. Moreover, in order to satisfy the penetration grade of 80/100 for asphalt, waste cooking oil (WCO) could be used with a dosage of only 2% at most [62]. However, for the source of palm kernel oil polyol, 20% bio-oil promoted the enhancement of asphalt binder penetration, whereas a higher content of 40% and 60% bio-oil decreased its penetration. Meanwhile, it was demonstrated that the penetration of bio-asphalt with 20% palm kernel oil polyol-based bio-oil was within the 80/100 penetration grade [63].

#### 4.1.2. Softening Point

Softening point refers to the temperature at which asphalt binder softens and reaches a lower viscosity under certain experimental conditions. It is related not only to the structure of the binder but also to its molecular weight. Similarly, as shown in Table 5, for most bio-oils, such as from waste wood, palm kernel oil polyol, waste cooking oil, and soybean, their addition lowered the softening point of asphalt binders [20,61,62,63]. Moreover, test results attained by Alamawi et al. revealed that the softening point values of bio-asphalts matched the standard value for petroleum asphalt 80/100# [63]. In particular, according to a study by He et al. the bio-asphalt had a slightly higher softening point than Styrene Butadiene Styrene (SBS) modified asphalt [60]. Similarly, the modification using pine wood-based bio-oil also increased the softening point of asphalt binder [64].

#### 4.1.3. Ductility

Ductility is an important index for evaluating the plasticity of binder. The greater the ductility, the better the plasticity of binder. Generally, the addition of bio-oils improves the plasticity of asphalt. For example, according to He et al., two kinds of bio-oils with the same constituents of 30% increased the ductility of SBS modified asphalt from 19.8 cm to 54.2 cm and 38.8 cm, respectively [60]. Similarly, soybean-based bio-oil with 10–30% contents also significantly promoted ductility [61].

#### 4.1.4. Penetration Index

Penetration index (PI) is often utilized to characterize the sensitivity to temperature of asphalt binder. A higher PI value of asphalt binder means lower susceptibility to temperature [65]. The properties of bio-oils affecting the temperature sensitivity of asphalts are different depending on the biomass sources. As observed in Table 5, the waste cooking oil (WCO)-based bio-asphalts had lower penetration index values than petroleum asphalt, indicating higher susceptibility to temperature [62]. However, for soybean bio-oil, its addition increased the penetration index values of asphalt binders. This indicates that the soybean bio-oil added to the reduction in temperature sensitivity of petroleum asphalt [61].

Accordingly, in comparison with petroleum asphalt, bio-asphalts exhibited different properties on penetration, softening point, ductility, and penetration index. The conventional properties of bio-asphalts were affected by the type of biomass source as well as bio-oil concentration. However, for most bio-oils, their addition softened asphalt by increasing the penetration and lowering the softening point, and they increased the plasticity. These influences may contribute to the improvement of workability of asphalt to a certain extent but decrease the rutting resistance.

### 4.2. Rheological Properties

#### 4.2.1. Viscosity and Workability

Viscosity is a somatic scale that determines the confrontation to the flow of binder, and it has a strong relationship with mixability and workability. Viscosity that is too high may contribute to the occurrence of thermal cracking at a truncated temperature of binder, while viscosity that is too reduced is more detrimental to high-temperature properties [66]. Generally, the viscosity of the asphalt is tested by Brookfield rotational viscometer. As shown in Table 6, Sun et al. tested the viscosities of waste wood bio-oil modified asphalts at temperatures 298 K, 333 K, 408 K, and 436 K. It was revealed that the bio-oil contributed to the decrease in viscosity of asphalt [67]. Fini et al. applied swine manure bio-oil as a modifier of petroleum asphalt, with a bio-oil dosage of 2 wt%, 5 wt%, and 10 wt%. Test results indicated that swine manure-based bio-oil improved the workability of the asphalt binder [68]. Similarly, Mills-Beale et al. found that the added swine manure bio-oil contributed to the decrease in viscosity of asphalt [32]. Moreover, Fini et al. explored the viscosities of four bio-asphalts containing swine manure bio-oil, corn stover bio-oil, miscanthus pellets bio-oil, and wood pellets bio-oil, respectively. In comparison, the miscanthus pellets-based bio-asphalt had the highest viscosity, while wood pellets-based bio-asphalt had the lowest viscosity [8]. Azahar et al. utilized untreated and treated waste cooking oils (WCOs) as modifiers of asphalt and found that both WCOs decreased the viscosity of asphalt [42]. Williams et al. performed the pyrolysis (PRL) process of the biomass materials oak wood, switchgrass, and corn stover and evaluated the properties of modified asphalts containing 3 wt%, 6 wt%, and 9 wt% bio-oils. When contrasted to petroleum asphalt, bio-asphalt was shown to have similar temperature sensitivity. Bio-asphalt performed similarly to petroleum asphalt in that it was a viscoelastic substance. This facilitates the application of these bio-oils as petroleum asphalt additives [39].

#### 4.2.2. Rutting, Fatigue, and Thermal Cracking Resistance

Asphalt binder qualities such as rutting resistance, fatigue resistance, and thermal cracking resistance are all crucial, these properties of bio-oil modified asphalts were summarized, as shown in Table 7. Sun et al. evaluated the properties of bio-asphalt at high temperature by rutting factor (G*/sin). Furthermore, a greater phase angle (δ), a smaller complex dynamic (G), and a lower fatigue factor (G*.sinδ) suggest superior low-temperature qualities. The test results indicated that waste wood bio-oil had a negative contribution to the high-temperature properties of asphalt but may improve its low-temperature cracking resistance [67]. Wen et al. utilized non-recoverable creep compliance (Jnr), performance grade (PG), failure strength, and critical strain energy density (CSED) in order to assess the properties of waste cooking oil modified asphalts. Specifically, Jnr was obtained to study the contribution of binder to permanent deformation of the mixture. The outcomes showed that the added bio-oil decreased the high and low PG grades and rutting resistance of asphalt, whereas it increased the thermal cracking resistance [69]. Similarly, Wang et al., found that waste cooking oil also decreased the rutting resistance of asphalt but improved its fatigue resistance according to the multiple stress creep recovery (MSCR), linear amplitude sweep (LAS), and elastic recovery (ER) tests [70]. Tang et al. studied the qualities of modified asphalts, including oak wood, switch grass, and maize stover bio-oils with amounts of 3%, 6%, and 9%. The results indicated that the performance grade of bio-asphalts varied with biomass sources and types of base asphalt [71]. Fini et al. and Mills-Beale et al. found that the cracking resistance of asphalt at low temperature was enhanced with the added swine manure bio-oil [32,68]. Nevertheless, according to Mills-Beale et al.’s study, the swine manure-based bio-asphalt also showed enhanced rutting resistance at higher temperatures [32]. Moreover, by comparing the rutting factors (G*/sinδ) of four bio-asphalts, it was found that swine manure-based bio-asphalt had the largest G*/sinδ, indicating the best rutting resistance, whereas wood pellets had the lowest rutting resistance [8]. Zhang et al. studied the effect of wood plant-based bio-oil, paraffinic oil, aromatic oil, and motor oil on rheological performance of petroleum asphalt. All bio-oils were shown to have a good influence on the low temperature and fatigue properties of asphalt but were shown to have an unfavorable influence on the high temperature characteristics [13,72]. Tu et al. obtained the segregation rate Rs value by rheological index of G*/sinδ in order to assess the storage stability of bio-asphalt at high temperatures. According to test results, it was recommended that the content of bio-oil be less than 20% by weight to retain a better thermal storage stability of asphalt [64]. Cao et al. studied the high temperature rheological properties of sawdust-based bio-asphalts with the DSR test. When the bio-oil was added into petroleum bitumen 50#, the rutting factor G*/sinδ was significantly decreased, indicating a decrease in high temperature deformation resistance. However, because of the bio-char contained in bio-oil, the addition of bio-oil into petroleum asphalt 70# increased its deformation resistance [53]. Guarin et al. experimented with bitumen modification using fish oil and rapeseed oil. The researchers concluded that bio-oil modification improved asphalt’s low-temperature qualities while reducing its high-temperature qualities. Furthermore, while these two bio-asphalts failed to meet Superpave Standards, the fish oil-modified asphalt binder outperformed the rapeseed oil-modified bitumen [73].

#### 4.2.3. Anti-Ageing Property

**Figure 4:**
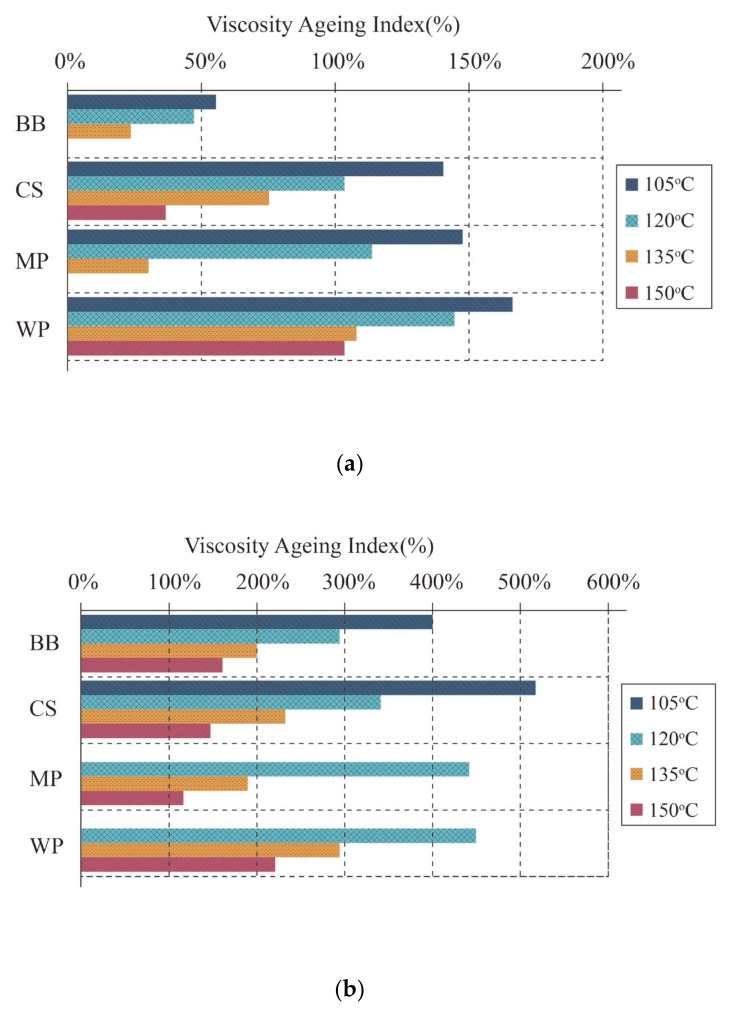
Viscosity ageing index of RTFO-aged and PAV-aged bio-oil modified asphalts: (**a**) RTFO-aged and (**b**) PAV-aged [8]. Note: BB refers to swine manure-based bio-asphalt; CS refers to corn stover-based bio-asphalt; MP refers to miscanthus pellets based bio-asphalt; WP refers to wood pellets-based bio-asphalt.

**Figure 5:**
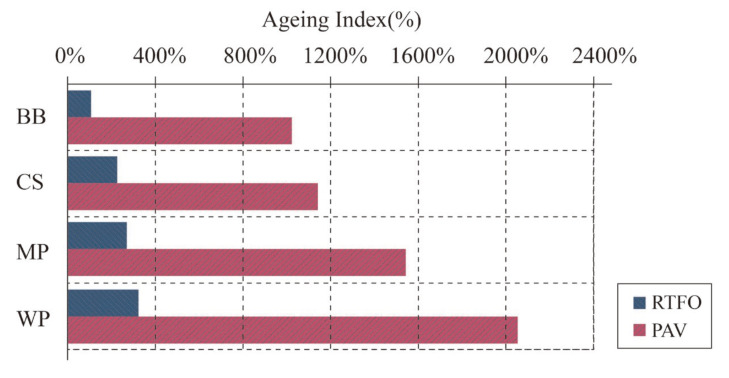
Ageing index for complex modulus of RTFO-aged and PAV-aged bio-oil modified asphalts at 64 °C and 10 rad/s [8]. Note: BB refers to swine manure-based bio-asphalt; CS refers to corn stover-based bio-asphalt; MP refers to miscanthus pellets-based bio-asphalt; WP refers to wood pellets-based bio-asphalt.

The chemical composition and structure of asphalt will alter because of the cumulative impacts of environmental elements such as elevated temperatures, oxygen, moisture, and UV radiation throughout the usage of asphalt materials. Their physical qualities, such as hardening, stickiness, brittleness, and so on, will alter as a result. Ageing is the term for these changes and events, and the core of asphalt ageing is the change in its physical qualities or chemical structure. Anti-ageing property of various bio-oil modified asphalts are shown in Table 8. According to Wen et al. and Azahar et al., adding waste cooking oil to asphalt lowered its anti-ageing properties by increasing percent mass loss and reducing the ageing index [42,69]. Fini et al. derived the viscosity ageing index (VAI) and ageing index (AI) based on the change in viscosity and complex shear modulus pre and post ageing in order to study the effects of ageing on the viscosity and complex shear modulus of four types of bio-asphalts, shown in Figure 4 and Figure 5. Higher VAI and AI values indicate worse anti-ageing property. It was found that the swine manure-based bio-asphalt had lower VAI values and AI values than that of plant-based bio-asphalts, indicating better anti-ageing property. This may be related to the molecular contribution of swine manure with petroleum asphalt, preventing hydrocarbons from interacting with oxygen. By comparing three kinds of plant-based bio-asphalts, the modified asphalt containing corn stover bio-oil had the best anti-ageing property, followed by the miscanthus pellets-based bio-asphalt and wood pellets-based bio-asphalt [8]. According to a study by Guarin et al., the percent mass loss of binder increased after RTFO ageing after bio-oil modification [73]. Wang et al. investigated the Jnr, Nf, and ER values of WCO-based bio-asphalts before and after ageing. It was found that bio-asphalts had higher ageing susceptibility and lower anti-ageing property than petroleum asphalt [70]. Similar conclusions were also obtained in other previous studies for waste wood, algae, swine manure, nanoalgae, switchgrass, and pine wood-based bio-asphalts [15,74,75].

According to the preceding investigations, the characteristics of bio-oil modified asphalt vary significantly depending on the biomass source. The majority of bio-oils help with workability, fatigue resistance, and low-temperature performance, whereas the high temperature and anti-ageing qualities of asphalt are reduced with the addition of bio-oil. In particular, swine manure-based bio-asphalts outperform planted-based bio-asphalts in terms of anti-ageing effectiveness. This may be largely determined by the chemical properties of bio-oils and bio-asphalts. Therefore, the influence of various bio-oils on the chemical fractions, functional groups, and molecular weight distribution of asphalt is summarized and discussed in the following section.

### 4.3. Chemical Properties

#### 4.3.1. Chemical Fractions

The modification by bio-oils had impacts on four chemical constituents of asphalt: saturates, asphaltenes, resins, and aromatics. Sun et al. discovered that the auxiliary waste wood bio-oil increased the content of saturates and aromatics of asphalt [67]. Zhang et al. found that the waste wood bio-oil increased aromatics content and resins content by 39.3% and 93.2%, respectively. This could explain the worse ageing resistance of asphalt and its higher adhesion to aggregates. However, adding bio-oil lowered saturates content in asphalt from 14.3% to 6.8%, as shown in Table 9 [21]. Similarly, according to the study by Guarin et al., due to the addition of ethyl ester from fish oil and fatty acid methyl esters (FAME) from rapeseed oil, there was a significant change in chemical components of bitumen. In particular, after modification by fish oil and rapeseed oil, the quantity of resins increased approximately 5% and 8%, respectively. The higher resin content resulted in the reduction in penetration index and intensification of viscosity of asphalt binder [73]. Wang et al. found that the WCO decreased the saturates contents and increased the contents of the resin of asphalt. When the WCO dosage was less than 3%, its addition also decreased the amount of asphaltenes. The bio-asphalts were more sensitive to ageing, which caused a shift from light components to asphaltenes [70]. Based on a study by Dhasmana et al., test results showed a significant difference in saturates, asphaltenes, resins, and aromatic (SARA) fractions between swine manure-based bio-asphalt and algae-based bio-asphalt. The added swine manure bio-oil evidently increased the amount of asphaltenes and resins by 48.8% and 92.8%, while it greatly decreased the aromatics amount from 54% to 13.1%. In contrast, for the algae-based bio-oil, its addition greatly decreased the amount of resins and asphaltenes but increased the aromatics content. This can explain to a certain extent why the swine manure-based bio-asphalt had better anti-ageing property than plant-based bio-asphalts [74].

#### 4.3.2. Functional Groups

In order to determine the functional groups in asphalt binders, Fourier transform infrared spectroscopy (FTIR) apparatus has also been frequently used. Yang et al. discovered that adding waste wood bio-oil to petroleum asphalt binder increased the ageing of the binder, which was not desired for the long-term performance of asphalt mixes [19]. Dong et al. came to the same conclusion that bio-asphalt’s anti-ageing properties were undesirable based on FTIR analysis peaks at 1700cm^−1^ (carbonyl) and 1030 cm^−1^ (sulphoxide linkages) [76]. Mills-Beale et al. investigated the chemical bond development of swine manure-based bio-asphalt using FTIR spectra. The swine manure bio-oil did not affect the functional groups of PG 64-22, but it could diminish the asphalt binder’s molecular carbonyl and sulphoxide ageing indices, suggesting a reduction in stiffness [32]. Zhang et al. studied the mechanics of using waste wood bio-oil to modify asphalt. Based on FTIR spectra analysis, chemical reactions occurred between bio-oil and petroleum asphalt, such as the reaction between phenol and formaldehyde and the reaction between alkanes and oxygen. Therefore, it is deduced that the properties of bio-asphalt were significantly affected by the type of bio-oils and the interaction mechanism between bio-oil and asphalt [21].

#### 4.3.3. Molecular Weight Distribution

The molecular weight distribution of asphalt binders is frequently investigated using gel permeation chromatography (GPC). Zhang et al. used GPC tests on petroleum asphalt, bio-oil, and bio-asphalt to observe how waste wood bio-oil altered the molecular weight distribution of asphalt. The results indicated that following bio-oil modification, the average molecular weight of asphalt decreased, and some large molecules decreased in size. This might explain why bio-asphalt has a lower anti-ageing property and a higher low-temperature fracture resistance [21]. Wang et al. studied the chemical characteristics of WCO-based bio-asphalt under oxidative ageing conditions by the GPC test. It was found that oxidation ageing increased the concentration of large molecules, and bio-asphalt was more susceptible to ageing than petroleum asphalt [70]. In comparison with petroleum asphalt, lignocellulose-based bio-oil had a lower weight-average molar mass of which macromolecular content was less and had smaller molecular structures [77].

## 5. Performance Assessment of Bio-Oil Modified Asphalt Mixtures

Asphalt mixture is a construction material consisting of asphalt binder, coarse aggregate, fine aggregate, mineral powder, and additional agents, among other things. Although the asphalt binder is critical to the mixture’s performance, additional elements have an impact on some aspects of the mixture’s performance. As a result, the performance evaluation of asphalt mixes incorporating bio-oils is important, and many researchers have looked into it. The influence of various bio-oils on the performance of asphalt mixtures is summarized in Table 10.

Mohammad et al. assessed the performance of modified asphalt mixture, including pine wood bio-oil with amounts of 20–50%. The results showed that adding bio-oil increased the fracture performance and moisture resistance of mixes at low temperatures. The bio-oil had no influence on the rutting resistance of the asphalt mixes, but it did reduce the intermediate temperature cracking resistance [78]. Yang et al. looked into the impact of waste wood bio-oil on asphalt mixture performance. Adding bio-oil greatly improved the fatigue properties of mixes, according to the findings. On the other hand, adding bio-oil had a detrimental effect on the rutting resistance of the mixes. It was suggested that the bio-oil amount should be controlled to be less than 10% [3]. Zhang et al. studied the impact of bio-based refined waste oils on asphalt mixture performance at low temperatures. The findings showed that bio-oils improved the low-temperature capabilities of binder and that the fracture temperatures of modified mixes were lower than those of normal mixtures [13]. Zeng et al. investigated the performance of castor oil-based modified asphalt mixture. The qualities at high temperatures and water resistance declined as the bio-oil amount increased but could meet the standard requirements, provided the bio-oil dosage was kept within a specified range [79]. Zhang et al. looked into the impact of waste wood bio-oil on the road performance of SBS modified asphalt mixtures and discovered that adding bio-oil increased moisture resistance but decreased high-temperature stability. Adding bio-oil also enhanced the adherence of bio-asphalt to aggregates at low temperatures [2]. According to a study by Gaudenzi et al., adding wood-based bio-oil to mixes increased their performance at low temperature but had no influence on fatigue resistance or moisture susceptibility [80]. Mirhosseini et al., discovered that when asphalt mixes were modified with sate seed oil, rutting resistance decreased but fatigue resistance increased. The moisture susceptibility of mixes was influenced just a little [43]. Nevertheless, Dong et al. discovered that the corn-based bio-oil enhanced asphalt mixture’s rutting resistance while somewhat lowering its cracking resistance at low temperature. Furthermore, the use of bio-oil reduced moisture resistance substantially [76].

In summary, the influences of bio-oils on the efficiencies of asphalt mixtures showed differences with various biomass sources, especially in intermediate temperature performance and moisture susceptibility. However, most bio-oils had an unfavorable impact on mixtures’ high temperature performance but exhibited favorable influence on their low-temperature performance. This deduction is consistent with the above outcomes about the properties of bio-asphalt binders.

## 6. Performance Optimization of Bio-Oil Modified Asphalt Binders/Mixtures

As summarized above, most bio-oil modified asphalts had better low and intermediate temperature performance but exhibited worse performance at high temperature. Thus, many scholars focused on the performance improvement of bio-asphalts, such as the high-temperature performance and anti-ageing property. Onochie et al. mixed 2% nano-clay and 4% nano-silicon into a swine manure-based bio-asphalt. It was found that nanomaterials increased the anti-ageing and high temperature capabilities of bio-asphalts, according to test results [81]. Raouf and Williams investigated the rheological characteristics of bio-oils made from switch grass, oak wood, and maize stover using three modifiers: two polyethylenes and an oxidized polyethylene. These polymers are thought to have aided in the enhancement of the high-temperature stability and ageing resistance of bio-oils [82]. Rubber powder was mixed with bio-oil modified asphalt by Peralta et al. The chemical compositions and PG grade of the rubber-modified bio-asphalts were examined after they were made. Rubber powder was discovered to be compatible with bio-asphalt. The PG grade of bio-asphalt could be tuned to PG 58-22 and PG 64-22 when the rubber powder concentration was 10% and 15% [83]. Fini et al. used crumb rubber together with swine manure for the modification of petroleum asphalt. The bio-oil improved the asphalt binder’s low-temperature qualities and workability, whereas crumb rubber improved the binder’s high-temperature characteristics [84]. Huang et al. used alcohol to accomplish esterification of bio-oil modified asphalt. It was discovered that adding 10% glycerol to the total binder by weight improved the modified asphalt’s characteristics at high and low temperatures, as well as its moisture susceptibility. Ageing, on the other hand, had a considerable influence on the performance at low temperature and moisture susceptibility of modified asphalt mixture [85]. Sun et al., endeavored to enhance the anti-ageing properties of asphalt treated using waste cooking oil. According to the study, it was better to use WCO, resin, hard asphalt particles, low-density polyethylene particles, and 4% SBS together in a certain mass ratio. The best bio-asphalt outperformed petroleum asphalt PEN 70 in terms of ageing resistance [86]. Zhang et al. attempted to increase the high temperature capabilities and ageing resistance of bio-asphalt by combining SBS modifier with waste wood bio-oil. The SBS content was set at 1% by weight, and the bio-oil amounts were set at 5%, 10%, 15%, and 20%. The SBS modified bio-asphalt was reported to have lower sensitivity to temperature and comparable or superior high-temperature performance compared to petroleum asphalt with penetration grade 50 [12]. Sun et al. increased the rutting resistance and fatigue resistance of asphalt treated by bio-oil by optimizing the bio-asphalt manufacturing process. This study employed leftover cooking oil as the bio-oil. The best manufacturing method was to combine the bio-oil, initiator, and acceleration solutions in a mass ratio of 100:1:2 for 2 h at 100 °C [87].

Generally, most researchers attempted to enhance the performance of bio-asphalts by combining various additional agents with bio-oils in the modification of petroleum asphalt binder. However, adding some agents, such as SBS and nanomaterials, increased the price of modified asphalt to some degree. Therefore, more reasonable optimization methods need to be proposed in order to promote the application of bio-asphalts in road engineering.

## 7. Conclusions

This paper provided a review on the characteristics of bio-oils and their utilization as additives of asphalts. The physico-chemical characteristics of various bio-oils were first reviewed. Then, the conventional, rheological, and chemical properties of bio-oil modified asphalt binders were discussed. The road performance of bio-asphalt mixtures was evaluated as well. Finally, performance optimization was summarized for bio-asphalt binders and mixtures. A few conclusions are drawn as follows:

(1) Bio-oils are derived from a wide range of biomass sources, and they are source-depending materials for which its properties vary with different sources. Bio-oils are extremely complex materials that contain various compounds. Compared to petroleum asphalts, bio-oils have significantly higher oxygen (O) element and oxygen-containing functional groups. That is an important reason why bio-oils are prone to ageing.
(2) Modification with bio-oils contributes to the improvement of workability, fatigue resistance, and low-temperature performance of asphalt. However, the high-temperature properties of asphalts weaken with the added bio-oils. After modification with bio-oils, some large molecules transform into small molecules in asphalts. Bio-asphalts are more sensitive to ageing, which causes a shift from light components to asphaltenes. In particular, swine manure-based bio-asphalts exhibit better anti-ageing property than planted-based bio-asphalts.
(3) The influences of bio-oils on the performance of asphalt mixtures differ with various biomass sources, especially in intermediate temperature performance and moisture susceptibility. Most bio-oils have a negative influence on the high-temperature performance of mixtures but exhibit favorable influence on their low-temperature performance.
(4) In order to improve the performance of bio-oil modified asphalts, most research has been limited to using additive agents together with bio-oils in order to modify petroleum asphalt, such as SBS and nanomaterials.

## 8. Recommendations for Future Work

Finding alternative uses for biomass energy has recently become a new development trend. Transferring waste biomass sources to bio-oils for use in road construction can help to minimize environmental pollution and fossil energy usage. Recent studies have indicated that bio-oils have the potential to partially replace petroleum asphalt in the application of pavement engineering. Although significant studies have been performed on the property evaluation of bio-oils and bio-asphalts, there are still many shortcomings and problems at present. Therefore, further study needs to be performed in order to understand the comprehensive performance and to solve current problems for promoting the large-scale application of bio-oils in practice.

(1) Since bio-oils are source-depending materials, their properties have great variability. It is necessary to propose reasonable evaluation indicators to roughly classify various bio-oils.
(2) A large number of studies have focused on the properties assessment of bio-asphalts, and few studies have been performed on their modification mechanism. Knowing the interaction mechanism between petroleum asphalt and bio-oils is helpful for better understanding the properties of bio-asphalts.
(3) The properties of bio-asphalts need to be further improved. Currently, most researchers are trying to add additive agents into bio-asphalts in order to enhance the high temperature properties, anti-ageing property, etc. However, applying some agents, such as SBS and nanomaterials, increases the price of modified asphalt to some degree. Therefore, more reasonable optimization methods need to be proposed in future. In particular, optimizing the properties of bio-oils themselves may be a better approach, and it is necessary to be studied.
(4) Further tests are needed to comprehensively understand the properties of bio-asphalts, such as thermal storage stability and their adhesion with aggregates. In addition, the long-term performance of bio-asphalt mixtures should be tested and tracked in field applications during the service life of pavement.

Such investigation will be meaningful for providing a more comprehensive theoret ical guidance for applying bio-oils in pavement engineering.

## Figures and Tables

**Table 1:** Physical characteristics of bio-oils [18,19,20,38,39,40].

Property	Characteristic Range
pH	2.5–3.5
Moisture dosage (wt %)	15–30
Viscosity (40 °C, cP)	35–1000
Viscosity (500 °C, cP)	40–100
Ash (wt %)	0–0.2

**Table 2:** Elemental composition of bio-oils.

Binders	Elemental Composition			
	C (%)	H (%)	O (%)	N (%)
Petroleum asphalt binder 1 [44]	83.17	10.28	1.33	0.45
Petroleum asphalt binder 2 [44]	85.20	10.30	1.25	0.58
Swine waste bio-oil [45]	72.58	9.76	13.19	4.47
Waste wood bio-oil [29]	54–56	5.5–7.2	35–45	0–0.2
Switchgrass bio-oil [46]	47.53	6.81	45.19	0.51
Alfalfa bio-oil [46]	53.88–56.84	8.47–7.86	31.3–32.73	3.73–4.59
Corn stover bio-oil [47]	46.5	5.9	46.2	--
Oakwood bio-oil [47]	60.5	6.5	34.6	--
Coffee residue bio-oil [35]	32.38	10.13	--	2.08
Laurel bio-oil [48]	49.65	8.1	41.63	5.0
Tea waste bio-oil [36]	69.26	8.97	15.58	6.19
Rapeseed [33]	72.8	10.8	13.1	3.3
Soybean [34]	67.89	7.77	--	10.84
Palm Shell bio-oil [49]	47.6	8.1	43.7	0.6
Rice straw bio-oil [50]	49.19	5.55	43.10	0.13
Bamboo sawdust bio-oil [50]	41.39	7.03	49.55	2.01
Delonix regia bio-oil [51]	50.62–58.32	6.93–7.65	32.75–41.34	1.06–1.28
Agricultural garbage-straw bio-oil [52]	59.27	12.68	27.25	0.80
Sawdust bio-oil [53]	68.550	7.176	22.219	0.110

**Table 3:** Functional groups of bio-oils.

Researchers	Binders	Functional Groups	Compounds
Zhang et al. [20]	Petroleum asphalt 70#	C-H bending S=O stretching CH_3_ bending C=C stretching C-H stretching	Aromatic compounds; sulfoxide; aliphatic compounds; alkanes
Zhang et al. [20]	Waste wood bio-oil	C-H bending S=O stretching C-O stretching CH_3_ bending −NO_2_ stretching C=C stretching C=O stretching C-H stretching O-H, N-H stretching	Aromatic compounds; sulfoxide; phenol and esters; aliphatic compounds; nitrogenous compounds; ketones, aldehydes, carboxylic acids, and acyls; alkanes; polymeric O-H, water, and NH_2_
Kawale et al. [51]	Delonix regia bio-oil	O-H stretching C-H stretching C=O stretching C=C stretching C-O stretching O-H bending C-H bending	Polymeric O-H, water impurities; alkanes, alkenes; ketones, aldehydes, and carboxylic acids; primary, secondary, tertiary alcohols, phenol, ester, and ether; aromatics
Shah et al. [54]	Spirogyra bio-oil	C-O stretching C-H bending C=C stretching C-H stretching O-H stretching	Ethers; alkanes; aromatics; alcohols
Cao et al. [53]	Sawdust bio-oil	O-H, N-H stretching C-H stretching C=O stretching C=C stretching C-H bending C-O stretching O-H stretching	Hydrocarbons; esters; aromatic hydrocarbons

**Table 4:** Chemical compounds in bio-oils.

Researchers	Biomass Sources	Processing Technology	Tested Main Compounds
Zhang et al. [21]	Waste wood	Fast pyrolysis	2-Methoxy-4-methylphenol; Naphthalene; 2-Methoxyphenol; Diethyl phthalate; Pentadecane; 2-Cyclopenten-1-one; Indene; 4-Ethyl-2-methoxyphenol
Kong et al. [57]	Ulva prolifera	Liquefaction over the parent HY catalyst	Benzene, 1-(1,1-dimethylethyl)-3,5-dimethyl-; 1*H*-Indole-3-carboxylic acid, 5-hydroxy-; Eicosanoic acid, 2-hydroxyethyl ester; 1-Propene, 1-chloro-; 2,2-Diethoxyacetophenone; Ethanone, 1-(2-benzothiazolyl)-; 9-Octadecenoic acid (Z)-, methyl ester; Benzonitrile
		Liquefaction over 8% Fe/HY catalyst	9-Octadecenoic acid (Z)-, methyl ester; Hexanoic acid, hexadecyl ester; 9,12-Octadecadienoic acid, methylester; Benzonitrie; 2-Amino-5-methylbenzoic acid; Hexadecanoic acid, methyl ester; Benzaladehyde, oxime, (Z)-; Benzene, [2-(1-propoxyethoxy)ethyl]-
Kawale et al. [51]	Delonix regia	Pyrolysis	Benzene; 4-Penten-2-one, 4-methyl-; 1,4-Butanediol, diacetate; *N,N,O*-Triacetylhydroxylamine; Pyridine; Propanedinitrile, (acetyloxy)methyl-; Acetohydroxamic acid; Pentane,2,2,4,4-tetramethyl-
Yuan et al. [52]	Agricultural garbage-straw	Liquefaction	2,4,6-Tris(1,1-dimethylethyl)-4-methylcyclohexa-2,5-dien-1-one and 1,2-Benzenedicarboxylic acid, butyl cyclohexyl ester.
Liu et al. [56]	Pine sawdust	Fast pyrolysis	l-polyglucose; Caproic acid; *o*-methoxyphenol; 2-hydroxy-3-methyl-2-cycloamylene ketone; furfural; 2-methoxy *p*-methylphenol; *p*-methyl phenol; 2-methoxy -2-amylene
Tahir et al. [55]	Jinan Pine waste	Fast pyrolysis	Acid Group; Carboxylic acids; Amide Group; Ether Groups; Phenolic Group
Shah et al. [54]	Spirogyra	Pyrolysis	3,4,5- Tri-methyl pyrazole; 2,3,4-Trimethyl-d-xylose; 1-*H* imidazole1,2,4,5-tetramethyl; 2-H imidazole,2,4,5-tetramethyl; Hexadecanenitrile; Benzonitrile, 4-methyl; 2-pyrrilidinone; 2-Hexadecene3,7,11,15-tetramethyl

**Table 5:** Conventional properties of bio-oil modified asphalts.

Researchers	Biomass Sources	Binders	Bio-Oil Content (%)	Penetration (25 °C) (0. 1 mm)	Softening Point (°C)	Ductility (5 °C) (cm)	Penetration Index (PI)
Zhang et al. [20]	--	Petroleum asphalt with penetration grade of 70	0	--	48	--	--
	Waste wood	Bio-asphalt	10	--	47.4	--	--
			15	--	47.2	--	--
			20	--	47	--	--
			25	--	46.9	--	--
			30	--	46.9	--	--
He et al. [60]	--	SBS modified asphalt	0	51.7	66.3	19.8	--
	Unknown	Bio-asphalt I	30	61.5	69.3	54.2	--
	Unknown	Bio-asphalt II	30	70.9	54	38.8	--
			50	78.8	51.7	Brittle failure	--
Alamawi et al. [63]	--	Petroleum asphalt with penetration grade of 80/100	0	85	46.5	--	--
	Palm kernel oil polyol	Bio-asphalt	20	98.7	40	--	--
			40	59.2	46	--	--
			60	70.5	45.5	--	--
Rasman et al. [62]	--	Petroleum asphalt with penetration grade of 80/100	0	86.8	41	--	−2.19
	Waste cooking oil	Bio-asphalt	1	92.1	39	--	−2.84
			2	95.6	40	--	−2.90
			3	116.1	38	--	−2.81
Li et al. [61]	--	Petroleum asphalt	0	62	48.5	0	−1.0
	Soybean	Bio-asphalt	10	64.1	50	6.8	−0.6
			15	83.1	48.9	6.1	−0.3
			20	108.6	46.3	9.7	−0.2
			30	172.8	45.4	10.7	1.3
Tu et al. [64]	--	Petroleum asphalt	0	76	48.7	>100 (15 °C)	--
	Pinewood	Bio-asphalt	5	--	50.5	--	--
			10	--	51.4	--	--
			15	--	51.7	--	--
			20	--	51.2	--	--

**Table 6:** Viscosity and workability of bio-oil modified asphalts.

Researchers	Base Binder	Biomass Sources	Bio-Oil Content (%)	Test Temperature	Results
Sun et al. [67]	Asphalt with penetration of 67.5	Waste wood	0,2.8	298, 333, 408, 436 K	Bio-oil decreased the viscosity of base asphalt.
Fini et al. [68]	Petroleum asphalt	Swine manure	2,5,10	--	Bio-oil improved the workability of asphalt binder.
Fini et al. [8]	Petroleum asphalt PG 64-22	Swine manure, corn stover, miscanthus pellets, wood pellets	10	105,120,135,150 °C	In comparison, the miscanthus pellets-based bio-asphalt had the highest viscosity, while wood pellets-based bio-asphalt had the lowest viscosity.
Azahar et al. [42]	Asphalt with 60/70 penetration grade	Waste cooking oil	3,4,5	135 °C	Waste cooking oil decreased the viscosity of asphalt.
Mills-Beale et al. [32]	Asphalt PG 64-22	Swine waste	5	12, 135, 150, 165, 180 °C	Bio-oil decreased the viscosity of asphalt binder.
Williams et al. [39]	Petroleum asphalt	Oak wood, switchgrass, corn stover	3,6,9	--	Bio-asphalts had similar temperature sensitivity to petroleum asphalt and behaved similar to viscoelastic materials.

**Table 7:** Rutting, fatigue, and thermal cracking resistance of bio-oil modified asphalts.

Researchers	Base Binder	Biomass Sources	Bio-Oil Content (%)	Test Equipment	Evaluation Index	Results
Sun et al. [67]	Asphalt with penetration depth of 67.5	Waste wood	0, 2.8, 5.5, 8.0, 10.4	DSR	G*/sinδ, G*, δ, G*sinδ	Bio-oil decreased the high temperature properties of asphalt but enhanced its low temperature cracking resistance.
Wen et al. [69]	Petroleum asphalt PG 58-28, PG 76-22, PG 82-16	Waste cooking oil	0, 10, 30, 60	DSR, BBR	Jnr, PG grade, failure strength, CSED	Bio-oil decreased the high and low PG grades and rutting resistance, whereas it increased the thermal cracking resistance.
Tang et al. [71]	Petroleum asphalt PG64-16, PG58-22, SBS modified asphalt	Oakwood, switchgrass, corn stover	3,6,9	DSR, BBR	High critical temperature, low critical temperature	The performance grade of bio-asphalts varied depending on biomass sources and types of base asphalt.
Fini et al. [68]	Petroleum asphalt	Swine manure	2,5,10	--	--	The bio-oil enhanced the cracking resistance of asphalt at low temperature.
Mills-Beale et al. [32]	Asphalt PG 64-22	Swine waste	5	DSR, BBR	G*, δ, G*/sinδ, creep stiffness, m-value	At higher temperatures, the bio-asphalt showed enhanced rutting resistance. Bio-oil enhanced the cracking resistance of asphalt at low-temperature.
Fini et al. [8]	Petroleum asphalt PG 64-22	Swine manure, corn stover, miscanthus pellets, wood Pellets	10	DSR	G*/sinδ	In comparison, swine manure bio-asphalt had the highest rutting resistance, whereas wood pellets had the lowest rutting resistance.
Zhang et al. [13,72]	Petroleum asphalt PG64-22	Wood plant, paraffifinic oil, aromatic oil, motor oil	5,6,7,10,11	DSR, BBR	G*/sinδ, PG grade, Jnr, stiffness, m value, G*sinδ,	All bio-oils had a favorable influence on the low temperature properties and fatigue resistance of asphalt, whereas they were detrimental to the high-temperature properties.
Tu et al. [64]	Petroleum asphalt AH-70	Pine wood	5,10,15,20	DSR	G*/sinδ, Rs	The content of bio-oil should be controlled to keep better thermal storage stability of asphalt.
Cao et al. [53]	Petroleum asphalt 50#, 70#	Sawdust	5,10,15,20	DSR	G*/sinδ, G*	Adding bio-oil into petroleum asphalt 50# decreased the high-temperature deformation resistance but improved the deformation resistance when applied in petroleum asphalt 70#.
Wang et al. [70]	Petroleum asphalt PG 64-22	Waste cooking oil (WCO)	1,3,5	DSR	Jnr, Nf, ER value	WCO decreased the resistance to rutting of asphalt but improved its fatigue resistance.
Guarin et al. [73]	Petroleum asphalt Pen 160/220	Fish oil, rapeseed oil	7,7.5,8	DSR, BBR	G*/sinδ, PG grade, stiffness, m value	Bio-oil modification enhanced the low-temperature performance of asphalt while diminishing its high-temperature performance. Fish oil-based bio-asphalt worked better than the rapeseed oil-based bio-asphalt.

**Table 8:** Anti-ageing property of bio-oil modified asphalts.

Researchers	Base Binder	Biomass Sources	Bio-Oil Content (%)	Test Equipment	Evaluation Index	Results
Wen et al. [69]	Petroleum asphalt PG 58-28, PG 76-22, PG 82-16	Waste cooking oil	0, 10, 30, 60	RTFO	Mass loss	The added bio-oil slightly increased the percent mass loss of asphalt.
Fini et al. [8]	Petroleum asphalt PG 64-22	Swine manure, corn stover, miscanthus pellets, wood pellets	10	RTFO, PAV, RV, DSR	Viscosity ageing index (VAI), ageing index (AI)	The swine manure-based bio-asphalt had better anti-aging property than plant-based bio-asphalts.
Guarin et al. [73]	Petroleum asphalt Pen 160/220	Fish oil, rapeseed oil	7,7.5,8	RTFO	Mass loss	After modification with bio-oil, the percent mass loss of asphalt increased after RTFO aging.
Wang et al. [70]	Petroleum asphalt PG 64-22	Waste cooking oil	1,3,5	RTFO, PAV, DSR	Jnr, Nf, ER value	Bio-oil lowered the anti-ageing property of asphalt.
Zhang et al. [15]	Petroleum asphalt	Waste wood	10,15,20,25,30	RTFO, Penetration, Softening point	Residual penetration ratio, a difference of softening point, mass loss	Bio-oil significantly decreased the anti-ageing property.
Azahar et al. [42]	60/70 penetration grade asphalt	Waste cooking oil (WCO)	3,4,5	RTFO, DSR	Aging index, G*/sinδ	WCO increased the temperature susceptibility of asphalt.
Dhasmana et al. [74]	Petroleum asphalt PG 64-22	Algae, swine manure, nanoalgae	--	RTFO, DSR	Complex modulus, phase angle	After ageing, all bio-asphalts behaved similar to elastic materials and exhibited significantly high modulus values.
Barzegari et al. [75]	Petroleum asphalt PG 64-22	Switch grass, pinewood	--	RTFO, PAV, DSR	Complex modulus, recovery capacity, R-value	The properties of bio-asphalts deteriorated significantly during long-term aging. Switchgrass bio-asphalt was affected the most.

**Table 9:** SARA fractions of bio-oil modified asphalts.

Researchers	Biomass Sources	Binders	Bio-Oil Content (%)	Saturates (%)	Asphaltenes (%)	Resins (%)	Aromatics (%)
Zhang et al. [21]	--	PG 64-22	0	14.3	24.4	8.8	42.7
	Waste wood	Bio-asphalt	15	6.8	16.7	17.0	59.5
Guarin et al. [73]	--	Bitumen Penetration Grade 160/220	0	9.0	49.6	22.4	19.0
	Fish oil	Bio-asphalt	--	7.6	44.3	27.2	20.9
	Rapeseed oil	Bio-asphalt	--	7.1	43.6	30.4	18.9
Wang et al. [70]	--	PG 64-22	0	19.5	18.2	22.5	38.2
		PG 64-22 (RTFO)		17.5	20.9	24.8	36.2
		PG 64-22 (PAV)		15.8	23.9	25.1	33.6
	Waste cooking oil	Bio-asphalt	1	14.4	16.3	27.6	37.3
		Bio-asphalt (RTFO)		14.1	19.2	24.6	37.5
		Bio-asphalt (PAV)		13.3	21.9	28.5	32.9
		Bio-asphalt	3	12.6	15.1	27.0	39.8
		Bio-asphalt (RTFO)		16.8	20.1	24.0	35.7
		Bio-asphalt (PAV)		18.0	17.4	26.5	34.0
		Bio-asphalt	5	15.0	18.9	29.5	32.2
		Bio-asphalt (RTFO)		15.6	20.1	26.9	32.7
		Bio-asphalt (PAV)		12.6	25.8	25.0	32.8
Dhasmana et al. [74]	--	PG 64-22	0	--	4.1	41.9	54
	Algae	Bio-asphalt	--	47	1.5	4.1	47.5
	Swine manure	Bio-asphalt	--	--	6.1	80.8	13.1

**Table 10:** The influences of bio-oils on the performance of asphalt mixtures.

Researchers	Biomass Sources	High-Temperature Performance	Intermedium Temperature Performance	Low-Temperature Performance	Moisture Resistance
Mohammad et al. [78]	Pinewood	Similar or improved	Decreased	Improved	Improved
Yang et al. [3]	Waste wood	Decreased	Significantly improved	--	--
Zhang et al. [13]	Wood plant liquid, refine waste oil	--	--	Improved	--
Zeng et al. [79]	Castor oil	Decreased	--	--	Decreased
Zhang et al. [2]	Waste wood	Decreased	--	Improved	Improved
Gaudenzi et al. [80]	Wood pulp and paper	--	Similar	Improved	Similar
Mirhosseini et al. [43]	Date seed oil	Decreased	Improved	--	Slightly affected
Dong et al. [76]	Corn	Improved	--	Decreased	Decreased

## Data Availability

Data supporting reported results can be found in publicly archived datasets.

## References

[1] Colbert B., Hasan M.R.M., You Z. (2016). A hybrid strategy in selecting diverse combinations of innovative sustainable materials for asphalt pavements. J. Traffic Transp. Eng. (Engl. Ed.).

[2] Zhang R., Wang H., Gao J., Yang X., You Z. (2017). Comprehensive Performance Evaluation and Cost Analysis of SBS-Modified Bioasphalt Binders and Mixtures. J. Mater. Civ. Eng..

[3] Yang X., You Z., Dai Q., Mills-Beale J. (2014). Mechanical performance of asphalt mixtures modified by bio-oils derived from waste wood resources. Constr. Build. Mater..

[4] Dinis-Almeida M., Afonso M.L. (2015). Warm mix recycled asphalt–a sustainable solution. J. Clean. Prod..

[5] Wang H., Zhang R., Chen Y., You Z., Fang J. (2016). Study on microstructure of rubberized recycled hot mix asphalt based X-ray CT technology. Constr. Build. Mater..

[6] Sun Z., Yi J., Huang Y., Feng D., Guo C. (2016). Properties of asphalt binder modified by bio-oil derived from waste cooking oil. Constr. Build. Mater..

[7] Azahar W.N.A.W., Bujang M., Jaya R.P., Hainin M.R., Mohamed A., Ngad N., Jayanti D.S. (2016). The potential of waste cooking oil as bio-asphalt for alternative binder—An overview. J. Teknol..

[8] Fini E.H., Oldham D., Buabeng F.S., Nezhad S.H. (2015). Investing the aging susceptibility of bio-modified asphalts. Airfield and Highway Pavements.

[9] Kim K.H., Bai X., Cady S., Gable P., Brown R. (2015). A quantitative investigation of free radicals in bio-oil and their potential role in condensed phase polymerization of cellulose-and lignin-derived pyrolysates. Underst. Thermochem. Convers. Biomass Overcome Biomass Recalcitrance.

[10] Miao X., Lin J., Bian F. (2020). Utilization of discarded crop straw to produce cellulose nanofibrils and their assemblies. J. Bioresour. Bioprod..

[11] Wei P.H., Xie R.H., Fu L., Zheng Y.J., Zhao H. (2021). Application Research on Crop Straw Biomass Waste in Logistics Packaging System. Advances in Artificial Intelligence and Security.

[12] Zhang R., Wang H., Gao J., You Z., Yang X. (2017). High temperature performance of SBS modified bio-asphalt. Constr. Build. Mater..

[13] Zhang L., Bahia H., Tan Y. (2015). Effect of bio-based and refined waste oil modifiers on low temperature performance of asphalt binders. Constr. Build. Mater..

[14] Yang X., You Z.-P., Dai Q.-L. (2013). Performance evaluation of asphalt binder modified by bio-oil generated from waste wood resources. Int. J. Pavement Res. Technol..

[15] Zhang R., Wang H., You Z., Jiang X., Yang X. (2017). Optimization of bio-asphalt using bio-oil and distilled water. J. Clean. Prod..

[16] Zhang X., Zhang K., Wu C., Liu K., Jiang K. (2020). Preparation of bio-oil and its application in asphalt modification and rejuvenation: A review of the properties, practical application and life cycle assessment. Constr. Build. Mater..

[17] Yang X., You Z., Mills-Beale J. (2014). Asphalt Binders Blended with a High Percentage of Biobinders: Aging Mechanism Using FTIR and Rheology. J. Mater. Civ. Eng..

[18] Ba T., Chaala A., Garcia-Perez M., Roy C. (2004). Colloidal Properties of Bio-Oils Obtained by Vacuum Pyrolysis of Softwood Bark. Storage Stability. Energy Fuels.

[19] Yang X., Mills-Beale J., You Z. (2017). Chemical characterization and oxidative aging of bio-asphalt and its compatibility with petroleum asphalt. J. Clean. Prod..

[20] Zhang R., Wang H., Jiang X., You Z., Yang X., Ye M. (2018). Thermal Storage Stability of Bio-Oil Modified Asphalt. J. Mater. Civ. Eng..

[21] Zhang R., Ji J., You Z., Wang H. (2020). Modification Mechanism of Using Waste Wood–Based Bio-Oil to Modify Petroleum Asphalt. J. Mater. Civ. Eng..

[22] Hill B., Oldham D., Behnia B., Fini E.H., Buttlar W.G., Reis H. (2016). Evaluation of low temperature viscoelastic properties and fracture behavior of bio-asphalt mixtures. Int. J. Pavement Eng..

[23] Yang X., You Z. (2015). High temperature performance evaluation of bio-oil modified asphalt binders using the DSR and MSCR tests. Constr. Build. Mater..

[24] Fini E.H., Hosseinnezhad S., Oldham D.J., Chailleux E., Gaudefroy V. (2016). Source dependency of rheological and surface characteristics of bio-modified asphalts. Road Mater. Pavement Des..

[25] Shao L., Wang H., Zhang R., Zheng W., Hossiney N., Wu C. (2021). Analysis of the chemical properties and high-temperature rheological properties of MDI modified bio-asphalt. Constr. Build. Mater..

[26] Peacocke G., Russell P., Jenkins J., Bridgwater A. (1994). Physical properties of flash pyrolysis liquids. Biomass Bioenergy.

[27] Bridgwater A. (1999). Principles and practice of biomass fast pyrolysis processes for liquids. J. Anal. Appl. Pyrolysis.

[28] Peacocke G., Bridgwater A. (1994). Ablative plate pyrolysis of biomass for liquids. Biomass Bioenergy.

[29] Zhang R., You Z., Wang H., Ye M., Yap Y.K., Si C. (2019). The impact of bio-oil as rejuvenator for aged asphalt binder. Constr. Build. Mater..

[30] Raouf M.A., Williams R.C. (2010). Rheology of fractionated cornstover bio-oil as a pavement material. Int. J. Pavements.

[31] Fini E.H., Kalberer E.W., Shahbazi A. (2011). Biobinder from swine manure: Sustainable alternative for asphalt binder. Proceedings of the Transportation Research Board 90th Annual Meeting, Washington, DC, USA, January, 2011.

[32] Mills-Beale J., You Z., Fini E., Zada B., Lee C.H., Yap Y.K. (2012). Aging Influence on Rheology Properties of Petroleum-Based Asphalt Modified with Biobinder. J. Mater. Civ. Eng..

[33] Onay O., Koçkar O.M. (2006). Pyrolysis of rapeseed in a free fall reactor for production of bio-oil. Fuel.

[34] Şensöz S., Kaynar I. (2006). Bio-oil production from soybean (*Glycine max* L.); fuel properties of Bio-oil. Ind. Crop. Prod..

[35] Chaiya C. Production of bio-oil from coffee residue using pyrolysis process. Proceedings of the World Congress on Engineering and Computer Science.

[36] Uzun B.B., Apaydin-Varol E., Ateş F., Özbay N., Pütün A.E. (2010). Synthetic fuel production from tea waste: Characterisation of bio-oil and bio-char. Fuel.

[37] Hill D.R., Jennings A.A. (2011). Bioasphalt from urban yard waste carbonization: A student study. (No. FHWA/OH-2011/13).

[38] Czernik S., Bridgwater A. (2004). Overview of applications of biomass fast pyrolysis oil. Energy Fuels.

[39] Williams R.C., Satrio J., Rover M., Brown R.C., Teng S. (2009). Utilization of fractionated bio oil in asphalt. Transportation Research Reord 3187.

[40] Özçimen D., Ersoy-Meriçboyu A. (2010). Characterization of biochar and bio-oil samples obtained from carbonization of various biomass materials. Renew. Energy.

[41] Lei Y., Wang H., Fini E.H., You Z., Yang X., Gao J., Dong S., Jiang G. (2018). Evaluation of the effect of bio-oil on the high-temperature performance of rubber modified asphalt. Constr. Build. Mater..

[42] Azahar W.N.A.W., Jaya R.P., Hainin M.R., Bujang M., Ngadi N. (2016). Chemical modification of waste cooking oil to improve the physical and rheological properties of asphalt binder. Constr. Build. Mater..

[43] Mirhosseini A.F., Tahami S.A., Hoff I., Dessouky S., Ho C.-H. (2019). Performance evaluation of asphalt mixtures containing high-RAP binder content and bio-oil rejuvenator. Constr. Build. Mater..

[44] Michalica P., Kazatchkov I.B., Stastna J., Zanzotto L. (2008). Relationship between chemical and rheological properties of two asphalts of different origins. Fuel.

[45] Fini E.H., Kalberer E.W., Shahbazi A., Basti M., You Z., Ozer H., Aurangzeb Q. (2011). Chemical Characterization of Biobinder from Swine Manure: Sustainable Modifier for Asphalt Binder. J. Mater. Civ. Eng..

[46] Mullen C., Boateng A.A. (2008). Chemical Composition of Bio-oils Produced by Fast Pyrolysis of Two Energy Crops. Energy Fuels.

[47] Metwally M., Williams R.C. (2010). Development of Non-Petroleum Based Binders for Use in Flexible Pavements (Final Report).

[48] Ertaş M., Alma M.H. (2010). Pyrolysis of laurel (*Laurus nobilis* L.) extraction residues in a fixed-bed reactor: Characterization of bio-oil and bio-char. J. Anal. Appl. Pyrolysis.

[49] Kim S.J., Jung S.H., Kim J.S. (2010). Fast pyrolysis of palm kernel shells: Influence of operation parameters on the bio-oil yield and the yield of phenol and phenolic compounds. Bioresour. Technol..

[50] Jung S.H., Kang B.S., Kim J.S. (2008). Production of bio-oil from rice straw and bamboo sawdust under various reaction conditions in a fast pyrolysis plant equipped with a fluidized bed and a char separation system. J. Anal. Appl. Pyrolysis.

[51] Kawale H.D., Kishore N. (2020). Pyrolysis of Delonix Regia and Characterization of Its Pyrolytic Products: Effect of Pyrolysis Temperature. J. Energy Resour. Technol..

[52] Yuan X., Xie W., Zeng G., Tong J., Li H. (2008). Influence of catalyst on the yields and properties of products from biomass liquefaction in subcritical water. Int. J. Biotechnol..

[53] Cao X., Liu Y., Cao X., Liu P., Miao C., Feng Y. (2019). Preparation and properties of biomass heavy oil and bio-asphalt. J. Chang. Univ. (Nat. Sci. Ed.).

[54] Shah Z., Cataluña Veses R., Silva R.D. (2016). GC-MS and FTIR analysis of bio-oil obtained from freshwater algae (spirogyra) collected from Freshwater. Int. J. Environ. Agric. Res..

[55] Tahir M.H., Cheng X., Irfan R.M., Ashraf R., Zhang Y. (2020). Comparative chemical analysis of pyrolyzed bio oil using online TGA-FTIR and GC-MS. J. Anal. Appl. Pyrolysis.

[56] Liu R., Wang H., Li T., Zhang C., Wu L. (2007). Production and characterisation of bio-oil from biomass fast pyrolysis in a fluidised bed reactor. Int. J. Glob. Energy Issues.

[57] Kong X., Wu S., Li X., Liu J. (2018). Microwave-Assisted Liquefaction of Ulva prolifera over Fe2O3-Modified HY Catalyst. J. Energy Eng..

[58] Capunitan J.A., Capareda S.C. (2013). Characterization and separation of corn stover bio-oil by fractional distillation. Fuel.

[59] Zeng M., Pan H., Zhao Y., Tian W. (2016). Evaluation of asphalt binder containing castor oil-based bioasphalt using conventional tests. Constr. Build. Mater..

[60] He M., Cao D.-W., Zhang H.-Y., Song Z.-R., Wu X.-W. (2015). Research on Conventional Performance of Modified Bio-Asphalt. J. Highw. Transp. Res. Dev. (Engl. Ed.).

[61] Li J., Zhang F., Liu Y., Muhammad Y., Su Z., Meng F., Chen X. (2019). Preparation and properties of soybean bio-asphalt/SBS modified petroleum asphalt. Constr. Build. Mater..

[62] Rasman M., Hassan N.A., Hainin M.R., Jaya R.P., Haryati Y., Shukry N.A.M., Abdullah M.E., Kamaruddin N.H.M. (2018). Engineering properties of bitumen modified with bio-oil. MATEC Web Conf..

[63] Alamawi M.Y., Khairuddin F.H., Yusoff N.I.M., Badri K., Ceylan H. (2019). Investigation on physical, thermal and chemical properties of palm kernel oil polyol bio-based binder as a replacement for bituminous binder. Constr. Build. Mater..

[64] Tu C., Chen Y.J., Cao D.W., He M. (2016). Thermal storage stability of bio-asphalt blended with petroleum asphalt. Highw. Traffic Technol. (Appl. Technol. Ed.).

[65] Read J., Whiteoak D. (2003). The Shell Bitumen Handbook, Shell Bitumen.

[66] Zaumanis M., Mallick R.B., Frank R. (2014). 100% recycled hot mix asphalt: A review and analysis. Resour. Conserv. Recycl..

[67] Sun B., Zhou X. (2018). Diffusion and Rheological Properties of Asphalt Modified by Bio-Oil Regenerant Derived from Waste Wood. J. Mater. Civ. Eng..

[68] Fini E.H., Yang S.-H., Xiu S. (2010). Characterization and Application of Manure-Based Bio-Binder in Asphalt Industry.

[69] Wen H., Bhusal S., Wen B. (2013). Laboratory Evaluation of Waste Cooking Oil-Based Bioasphalt as an Alternative Binder for Hot Mix Asphalt. J. Mater. Civ. Eng..

[70] Wang C., Xie T., Cao W. (2019). Performance of bio-oil modified paving asphalt: Chemical and rheological characterization. Mater. Struct..

[71] Tang S., Williams R.C. (2009). Antioxidant effect of bio-oil additive ESP on asphalt binder. Proceedings of the 2009 Mid-Continent Transportation Research Symposium, Ames, IA, USA, August, 2009.

[72] Zhang L., Bahia H., Tan Y., Cheng L. (2017). Effects of refined waste and bio-based oil modifiers on rheological properties of asphalt binders. Constr. Build. Mater..

[73] Guarin A., Khan A., Butt A.A., Birgisson B., Kringos N. (2016). An extensive laboratory investigation of the use of bio-oil modified bitumen in road construction. Constr. Build. Mater..

[74] Dhasmana H., Ozer H., Al-Qadi I.L., Zhang Y., Schideman L., Sharma B.K., Chen W.-T., Minarick M.J., Zhang P. (2019). Rheological and Chemical Characterization of Biobinders from Different Biomass Resources. Transp. Res. Rec. J. Transp. Res. Board.

[75] Barzegari S., Solaimanian M. (2020). Rheological behavior of bio-asphalts and effect of rejuvenators. Constr. Build. Mater..

[76] Dong Z., Zhou T., Luan H., Wang H., Xie N., Xiao G.-Q. (2018). Performance evaluation of bio-based asphalt and asphalt mixture and effects of physical and chemical modification. Road Mater. Pavement Des..

[77] Ding Y., Shan B., Cao X., Liu Y., Huang M., Tang B. (2021). Development of bio oil and bio asphalt by hydrothermal liquefaction using lignocellulose. J. Clean. Prod..

[78] Mohammad L.N., Elseifi M.A., Cooper S.B., Challa H., Naidoo P. (2013). Laboratory Evaluation of Asphalt Mixtures that Contain Biobinder Technologies. Transp. Res. Rec. J. Transp. Res. Board.

[79] Zeng M., Tian W., Zhu Y., Li J. (2017). Study on Performance of Castor Oil-based Bioasphalt Blended Asphalt Mixture. J. Hunan Univ. (Nat. Sci.).

[80] Gaudenzi E., Canestrari F., Lu X., Cardone F. (2021). Performance Assessment of Asphalt Mixture Produced with a Bio-Based Binder. Materials.

[81] Onochie A., Fini E., Yang X., Mills-Beale J., You Z. (2013). Rheological characterization of nano-particle based bio-modified binder. Proceedings of the Transportation Research Board 92nd Annual Meeting, January, 2013.

[82] Raouf M.A., Williams R.C. (2009). Determination of pre-treatment procedure required for developing bio-binders from bio-oils. Proceedings of the 2009 Mid-Continent Transportation Research Symposium, Ames, IA, USA, August 2009.

[83] Peralta J., Raouf M.A., Tang S., Williams R.C. (2012). Bio-Renewable Asphalt Modifiers and Asphalt Substitutes. Sustainable Bioenergy and Bioproducts.

[84] Fini E.H., Hosseinnezhad S., Oldham D., McLaughlin Z., Alavi Z., Harvey J. (2017). Bio-modification of rubberised asphalt binder to enhance its performance. Int. J. Pavement Eng..

[85] Huang H.L. (2015). Research on Road Performance of Bio-Asphalt Binder and Mixture.

[86] Sun D., Lu T., Xiao F., Zhu X., Sun G. (2017). Formulation and aging resistance of modified bio-asphalt containing high percentage of waste cooking oil residues. J. Clean. Prod..

[87] Sun Z., Yi J., Feng D., Kasbergen C., Scarpas A., Zhu Y. (2018). Preparation of bio-bitumen by bio-oil based on free radical polymerization and production process optimization. J. Clean. Prod..

